# Oncogenic transmembrane protein 158 drives the PI3K/Akt signaling pathway to accelerate gastric cancer cell growth

**DOI:** 10.1590/1414-431X2023e12943

**Published:** 2023-11-13

**Authors:** Xiaoting Cui, Jie Lu, Cuijuan Zhao, Yu Duan

**Affiliations:** 1 Department of Gastroenterology, The Third Affiliated Hospital of Inner Mongolia Medical University, Inner Mongolia Baogang Hospital, Baotou, China; 2 Department of Gastroenterology, The Fourth Affiliated Hospital of Baotou Medical College, Baotou Eighth Hospital, Baotou, China

**Keywords:** Gastric cancer, TMEM158, Proliferation, PI3K/AKT signaling

## Abstract

Gastric cancer (GC) is a serious threat to human health and an important cause of cancer-related death. Herein, we evaluated the influence of transmembrane protein 158 (TMEM158) on GC cell growth. According to Genomic Spatial Event (GSE) and The Cancer Genome Atlas (TCGA) databases, TMEM158 content is amplified in GC tissues. The diagnostic value of TMEM158 expression in GC is huge. GC sufferers with high expression of TMEM158 were associated with poor overall survival. In addition, TMEM158 content was increased in GC cells. TMEM158 promoted GC cell proliferation by modulating the PI3K/Akt signaling pathway. Lack of TMEM158 reduced GC tumor growth. Collectively, TMEM158 accelerated GC cell proliferation by modulating the PI3K/Akt signaling pathway, making it a prospective biomarker for survival in GC patients.

## Introduction

Gastric cancer (GC) is a malignant cancer that is frequently asymptomatic or presents with non-specific symptoms like indigestion in its early stages, being often ignored by patients (1). Furthermore, GC has a very high incidence worldwide, especially in East Asia (2). Although GC is well treated with surgery and adjuvant chemotherapy, most patients have a relatively poor prognosis (3,4). To make matters worse, more than 80% of patients are diagnosed at an advanced stage, missing the best time for treatment (5). Consequently, the discovery of new biomarkers is critical for early-stage diagnosis of GC.

Transmembrane protein 158 (TMEM158) has been proven to be an important cancer regulator. For instance, Cheng et al. (6) reported that TMEM158 level is increased in ovarian cancer. Silence of TMEM158 decreased ovarian cancer cell proliferation, cell adhesion, and cell invasion. In addition, Liu et al. (7) found that TMEM158 level is higher in colorectal cancer (CRC). Downregulation of TMEM158 repressed cell growth and migration, but improved apoptosis in CRC. Moreover, Li et al. (8) discovered that mRNA level of TMEM158 is associated with poor overall survival (OS) of glioma patients. Additionally, lack of TMEM158 attenuated proliferation, migration, and invasion of glioma cells, TMEM158 content was higher in laryngeal cancer, and TMEM158 may play a role in modulating cell apoptosis in laryngeal cancer (9). Nevertheless, the impact of TMEM158 in GC is uncertain.

PI3K/AKT signaling is associated with cell cycle, cell proliferation, cell invasion, and longevity (10). According to Xie et al. (10), PI3K/Akt signaling is linked with erythropoiesis. In addition, the PI3K/Akt pathway is indispensable to cell apoptosis, and exerts its effects in tumorigenesis (11). In addition, the PI3K/Akt signaling pathway participates in the modulation of cell behavior in prostate cancer (12). Moreover, Ediriweera et al. (13) found that the PI3K/AKT signaling pathway plays a key role in tumorigenesis and cell proliferation in ovarian cancer. In this research, we investigated the effect of PI3K/AKT signaling in GC cells.

## Material and Methods

### Data collection and analysis

The gene expression profiles for GC were investigated in publicly accessible Gene Expression Omnibus (GEO) database at NCBI (http://www.ncbi.nlm.nih.gov/geo/). Genomic Spatial Event (GSE) 54129, GSE103236, GSE79973, GSE19826, GSE13911, and GSE118916 were selected for this paper. This research collected TMEM158 data of 408 GC specimens and 31 normal specimens from TCGA (https://portal.gdc.cancer.gov). In addition, the TMEM158 data of 408 GC specimens and 211 normal specimens from TCGA-GTEx (GEPIA: http://gepia.cancer-pku.cn) were also used in this work. To assess the diagnostic value of TMEM158, we generated the receiver operating characteristic (ROC) curve utilizing the above data. In addition, cross-validation of biomarkers associated with survival in GC was performed using transcriptomic data of 1065 GC patients (Oncotarget, doi: 10.18632/oncotarget.10337).

### Cell lines and culture

In this research, human GC cell lines (MKN-45, SNU-1, NCI-N87, and AGS) were provided by Procell Biotechnology (China). Human gastric epithelial cell line (GES-1) was obtained from Chuan Qiu Biotechnology (China). AGS cells were grown in Ham's F-12 medium (Invitrogen, USA) with 5% CO_2_ at 37°C. MKN-45, SNU-1, NCI-N87, and GES-1 cells were grown in RPMI-1640 medium (Invitrogen) with 5% CO_2_ at 37°C.

### Cell transfection

The small interfering RNA (siRNA) targeting TMEM158 (si-TMEM158) and normal control (si-NC), TMEM158 overexpression plasmid (pcDNA-TMEM158) and control (pcDNA), short hairpin RNA (shRNA) binding TMEM158 (sh-TMEM158) and control (sh-NC), 740 Y-P (the activator of PI3K signaling), and LY294002 (the inhibitor of PI3K signaling) were obtained from Ribobio (China). Lipofectamine 3000 (Invitrogen) was applied to implement the transfection consistent with the instructions. With the intention of activating or inhibiting the PI3K/Akt signaling pathway, cells were treated with 740 Y-P (30 μM; Ribobio) or LY294002 (25 μM; Ribobio) for 24 h in prior to transfection.

### Western blot

The samples were lysed with RIPA buffer (Invitrogen) and the protein content was analyzed using a BCA kit (Solarbio, China). Protein was then separated on a 12% SDS-PAGE gel. Next, the disconnected protein bands were moved onto a PVDF membrane (Invitrogen). After blocking, the membrane was incubated overnight with primary antibodies at 4°C. The antibodies used were: anti-TMEM158 (PA5-49532; 1:1,000; Cell Signaling Technology, USA), anti-PI3K (AF5121; 1:600; Affinity Biosciences, China), anti-p-PI3K (AF4372; 1:1000; Affinity Biosciences), anti-p-Akt (ab18206; 1:1000; Abcam, USA), anti-Akt (ab8805; 1:1000; Abcam), anti-Ki67 (ab16667; 1:1000; Abcam), and anti-β-actin (BM3873; 1:2000; Boster Biological Technology, China). Subsequently, the membranes were treated with goat anti-rabbit IgG (BA1056; 1:5000; Boster Biological Technology) for 1 h. The protein blots were observed using ECL-Plus reagent (Invitrogen).

### Cell counting kit-8 (CCK8) assay

AGS and MKN-45 cells (2.0×10^3^/well) were seeded onto 96-well plates. After various transfections, CCK-8 solution (10 μL/well; Solarbio) was supplemented and incubated at 25°C for 1.5 h. The absorbance at 450 nm was identified through a microplate reader (Varioskan LUX; Thermo Fischer Scientific, USA).

### 5-Ethynyl-2'-deoxyuridine (EdU) assay

After various treatments, the AGS and MKN-45 cells were separately seeded onto 24-well plates. An EdU kit (Boster Biological Technology) was employed for appraising cell proliferation. Nuclei were exposed to EdU and DAPI (Boster Biological Technology). Finally, images were acquired via laser confocal microscopy (Leica, Germany).

### Tumor model

All BALB/c nude mice (4 weeks old; female) were acquired from Shanghai Laboratory Animal Company (SLAC, China). AGS cells were transfected with sh-TMEM158 or sh-NC. Then, AGS cells (2×10^6^) were injected into the mice (6 mice/group). Tumor volume was appraised every week using the formula: V (mm^3^) = (length × width^2^) / 2. All animals were sacrificed five weeks later and tumor weight was measured. Tumor tissues were used for other tests.

### Hematoxylin-eosin staining (HE)

The tumor tissues were exposed to 10% paraformaldehyde (Solarbio) for 48 h, embedded in paraffin (Solarbio), and paraffin slices with a thickness of 5 μm were prepared. Then, these paraffin slices were stained with HE (Solarbio), and morphological alterations were detected using a microscope (Leica).

### Immunohistochemistry (IHC)

Detection of Ki67 was carried out in 3-μm thick paraffin slices. These slices were incubated with primary antibodies anti-Ki67 (ab8805; 1:500; Abcam) at 4°C overnight. Subsequently, slices were exposed to HRP-conjugated secondary antibodies (BM3895, 1:500, Boster Biological Technology) at 37°C for 0.5 h. Finally, the slices were treated with DAB kit (Solarbio) for 5 min and observed using a microscope (Leica).

### Statistical analysis

Data are reported as means±SD. The Shapiro-Wilk test was used to verify the normality of the data. Comparisons were made by Student's *t*-test between two groups and by one-way analysis of variance (ANOVA) followed by *post hoc* Tukey's multiple comparisons test among multiple groups. Clinical factors associated with OS in GC patients were confirmed utilizing Cox regression and the Kaplan-Meier procedure (plot: http://kmplot.com/analysis). At least three repetitions were carried out in each test. Statistical analyses were conducted using the SPSS 22.0 software (SPSS Inc., IBM, USA). P<0.05 was considered to be statistically significant.

## Results

### Differentially expressed genes

**Figure 1:**
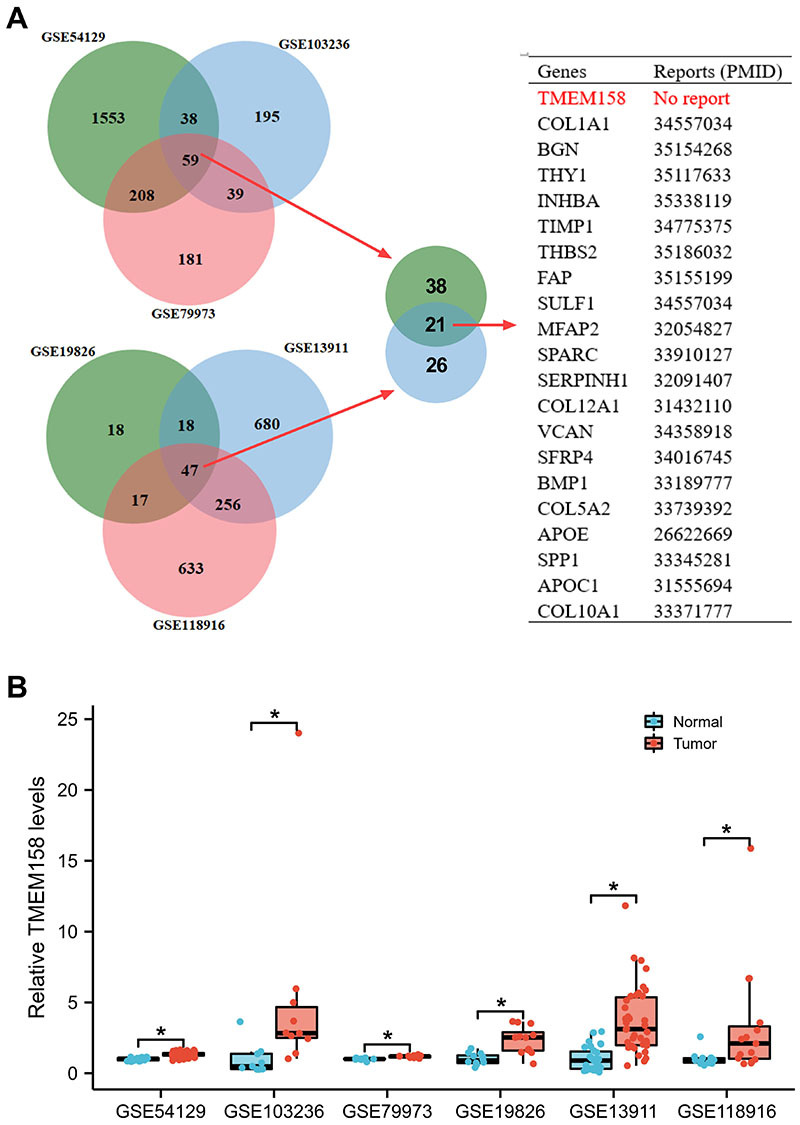
**A**, The Venn diagram shows overlapping genes in different Genomic Spatial Event (GSE) databases, and the table shows the research status of each gene. **B**, The TMEM158 level was increased in gastric cancer samples. Data are reported as medians and interquartile ranges. *P<0.05 (Student's *t*-test).

We found 59 genes that overlapped in GSE54129, GSE103236, and GSE79973 and 47 genes that overlapped in GSE19826, GSE13911, and GSE118916. There were 21 duplicated genes in the above 2 collections. Among them, only TMEM158 has not been studied yet, so it is the focus of this paper (Figure 1A). In the above 6 GSE databases, the content of TMEM158 was significantly increased in GC tumor tissues *vs* normal controls (Figure 1B). Taken together, these results indicated that TMEM158 was highly expressed in GC.

### Diagnostic value of TMEM158 expression in GC

**Figure 2:**
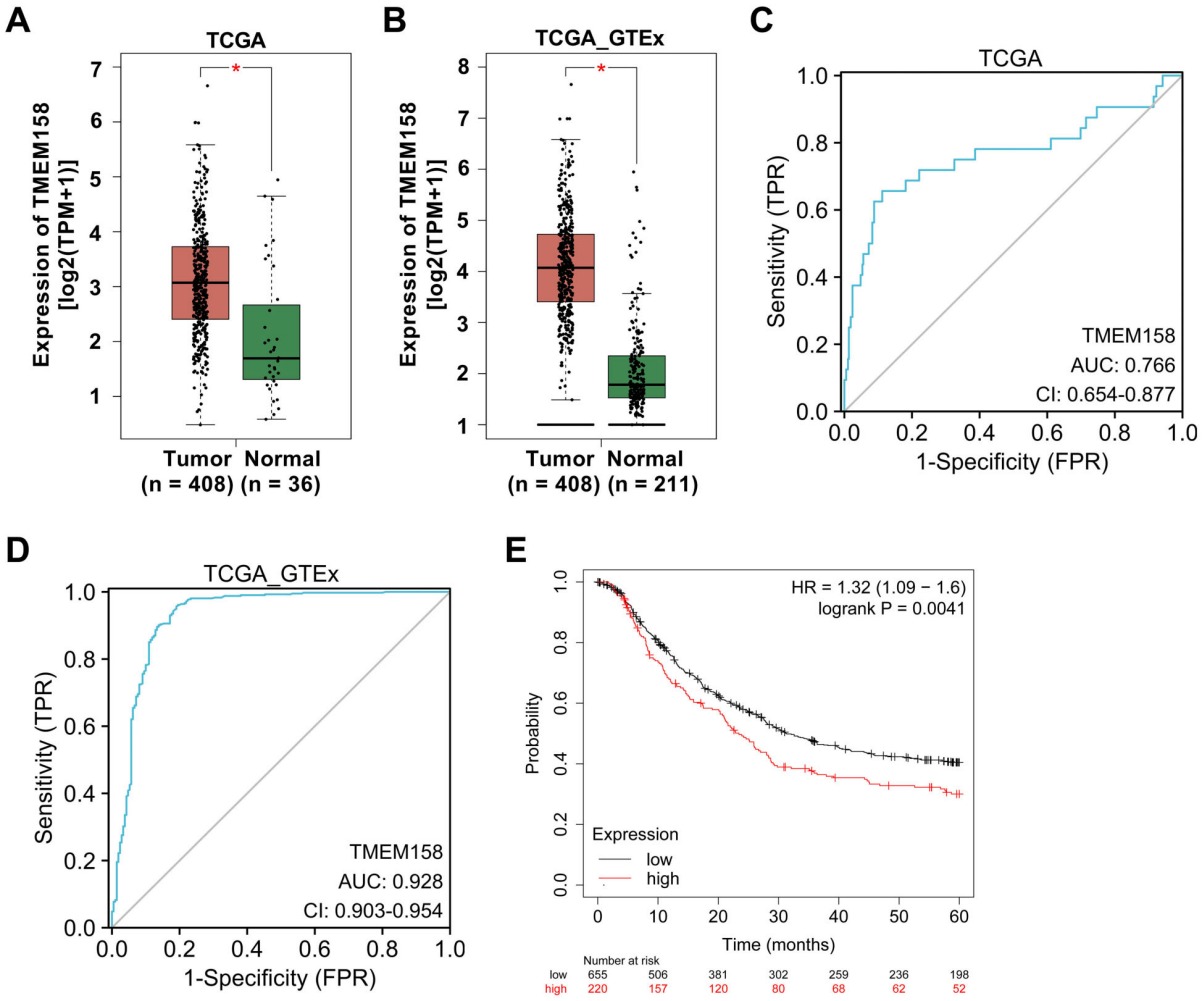
Diagnostic value of TMEM158 in gastric cancer (GC). **A** and **B**, TMEM158 expression in GC. **C** and **D**, ROC curves of TMEM158 for TCGA and TCGA-GTEx. **E**, Overall survival of GC patients with low or high level of TMEM158. Data are reported as means±SD. *P<0.05 (Student's *t*-test).

In this part, we confirmed the association between TMEM158 expression and GC diagnosis. According to the data of TCGA and TCGA-GTEx, the TMEM158 content was increased in GC tumor tissues compared with normal controls (Figure 2A and B). To appraise the diagnostic value of TMEM158, we created the ROC curve utilizing the data of TCGA and TCGA-GTEx. The areas under the ROC curve were 0.766 (95%CI: 0.654-0.877) (Figure 2C) and 0.928 (95%CI: 0.903-0.945) (Figure 2D), respectively, which indicated a good diagnostic value. As validated in Figure 2E, high expression of TMEM158 was associated with poor OS (P=0.0041; hazard ratio (HR): 1.32; 95%CI: 1.09-1.6) of GC patients. In brief, these outcomes indicated that the diagnostic value of TMEM158 expression in GC was significant, and TMEM158 was associated with OS of GC patients.

### TMEM158 promoted GC cell proliferation

**Figure 3:**
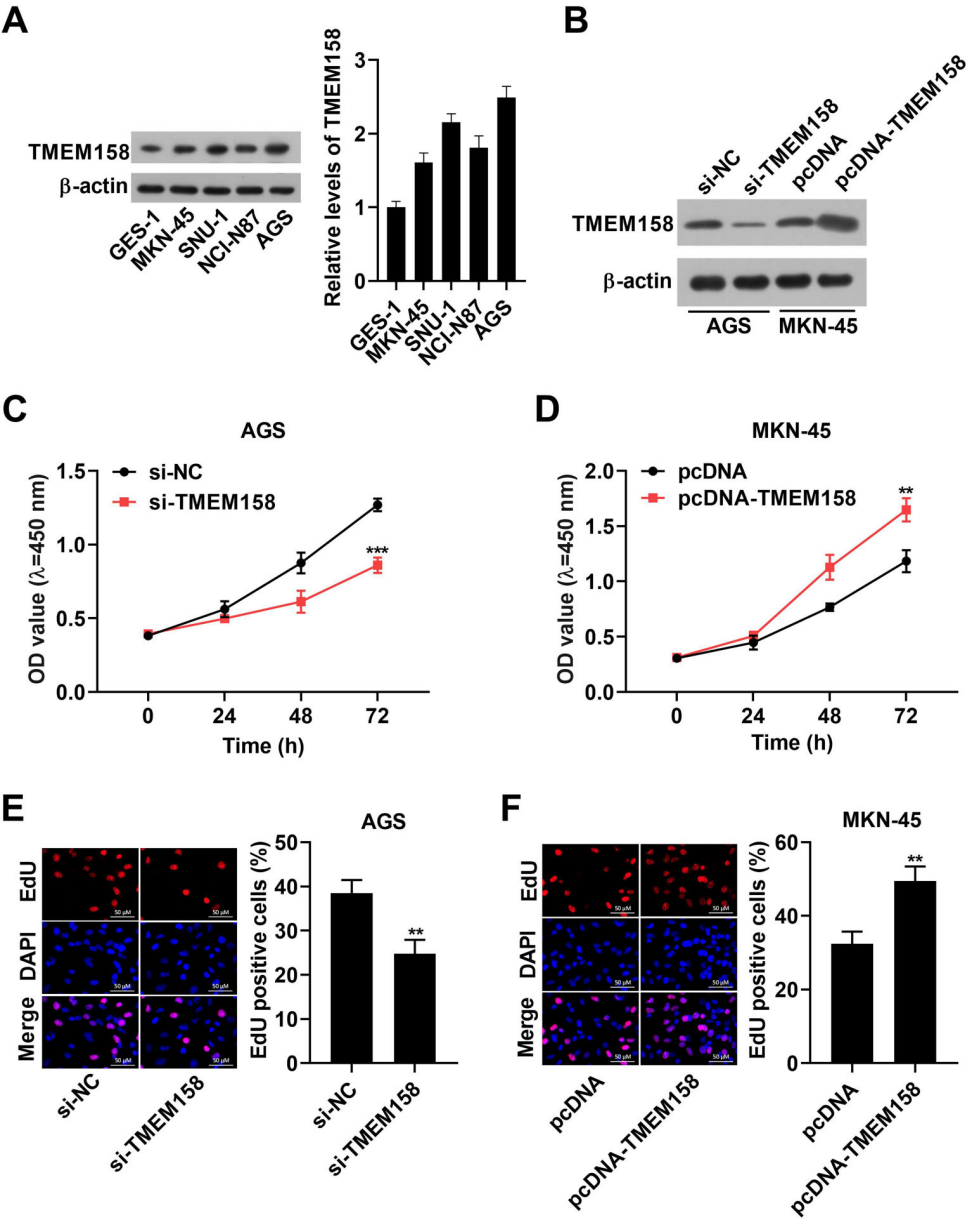
TMEM158 regulated gastric cancer (GC) cell proliferation. **A** and **B**, The TMEM158 content was monitored by western blot. **C** and **D**, Cell vitality was assessed by CCK8 assay in AGS and MKN-45 cell lines. **E** and **F**, Cell proliferation was examined in AGS and MKN-45 cell lines by EdU assay (scale bar 50 μM). Data are reported as means±SD. **P<0.01 and ***P<0.001 (Student's *t*-test). NC: normal control.

Primarily, TMEM158 was increased in GC cells (MKN-45, SNU-1, NCI-N87, and AGS) compared to GES-1 cells (Figure 3A). AGS and MKN-45 cells were selected for study in subsequent experiments. Furthermore, the content of TMEM158 was diminished by si-TMEM158 transfection in AGS cells, but increased after TMEM158 overexpression in MKN-45 cells (Figure 3B). In addition, cell proliferation was reduced by downregulation of TMEM158 in AGS cells (Figure 3C and E), but increased by overexpression of TMEM158 in MKN-45 cells (Figure 3D and F). These results indicated that TMEM158 contributed to GC cell proliferation.

### TMEM158 promoted activation of PI3K/Akt signaling

**Figure 4:**
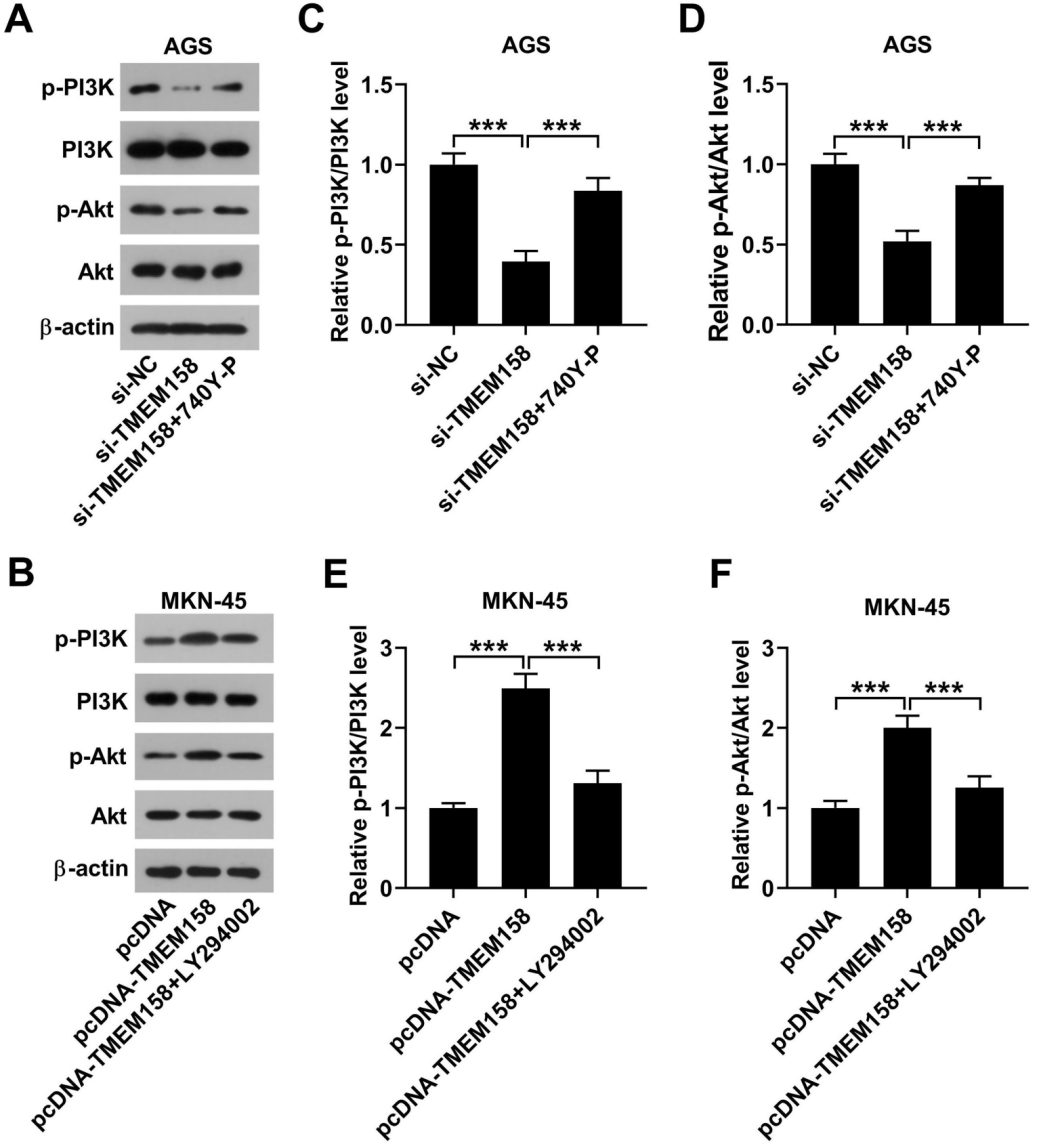
TMEM158 regulated activation of the PI3K/Akt signaling in AGS and MKN-45 cell lines. **A** and **B**, The activity of PI3K/AKT signaling was assessed by western blot. The level of p-P13K/P13K (**C** and **E**) and the level of p-Akt/Akt (**D** and **F**) are shown. Data are reported as means±SD. ***P<0.001 (ANOVA). NC: normal control.

Interestingly, the levels of p-P13K/P13K and p-Akt/Akt were reduced by downregulation of TMEM158, but this influence was eradicated by 740 Y-P co-treatment in AGS cells (Figure 4A, C, and D). Inversely, the levels of p-P13K/P13K and p-Akt/Akt were increased by overexpression of TMEM158, while this influence was eliminated by LY294002 co-treatment in MKN-45 cells (Figure 4B, E, and F). Therefore, we confirmed that TMEM158 accelerated activation of the PI3K/AKT signaling.

### PI3K activation or inhibition reversed the effect of TMEM158 silencing or overexpression

**Figure 5:**
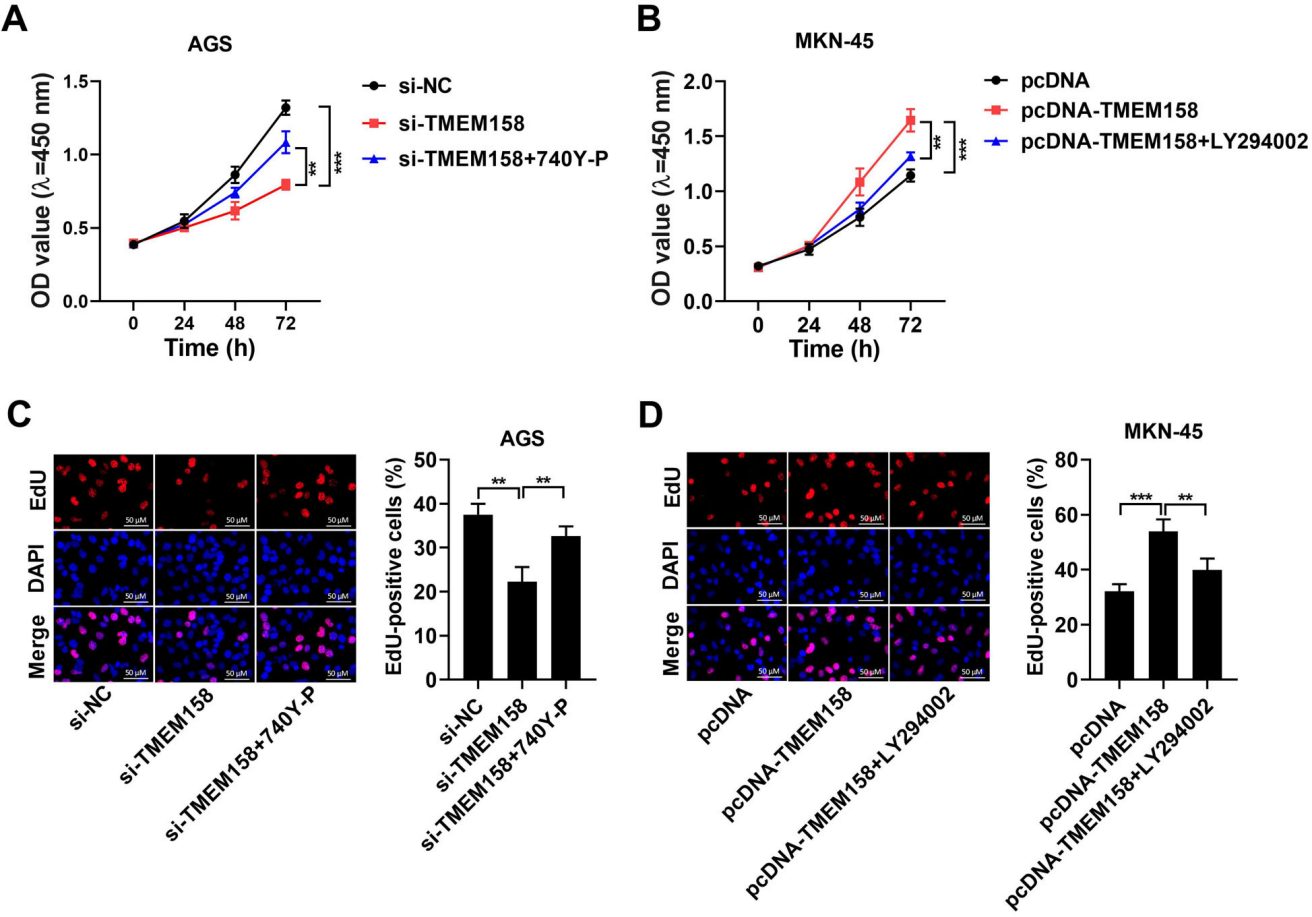
TMEM158 regulated gastric cancer (GC) cell proliferation by PI3K/AKT signaling. **A** and **B**, Cell viability was assessed by CCK8 assay in AGS and MKN-45 cell lines. **C** and **D**, Cell proliferation was examined in AGS and MKN-45 cell lines by EdU assay (scale bar 50 μM). Data are reported as means±SD. **P<0.01 and ***P<0.001 (ANOVA).

Cell proliferation was reduced by si-TMEM158 transfection, but this influence was reversed by 740 Y-P co-treatment in AGS cells (Figure 5A and C). Moreover, cell proliferation was enhanced by overexpression of TMEM158, but this influence was reversed by LY294002 co-treatment in MKN-45 cells (Figure 5B and D). Consequently, we established that PI3K activation or inhibition overturned the influence of TMEM158 silencing or overexpression on GC cell proliferation.

### TMEM158 silencing reduced GC tumor growth

**Figure 6:**
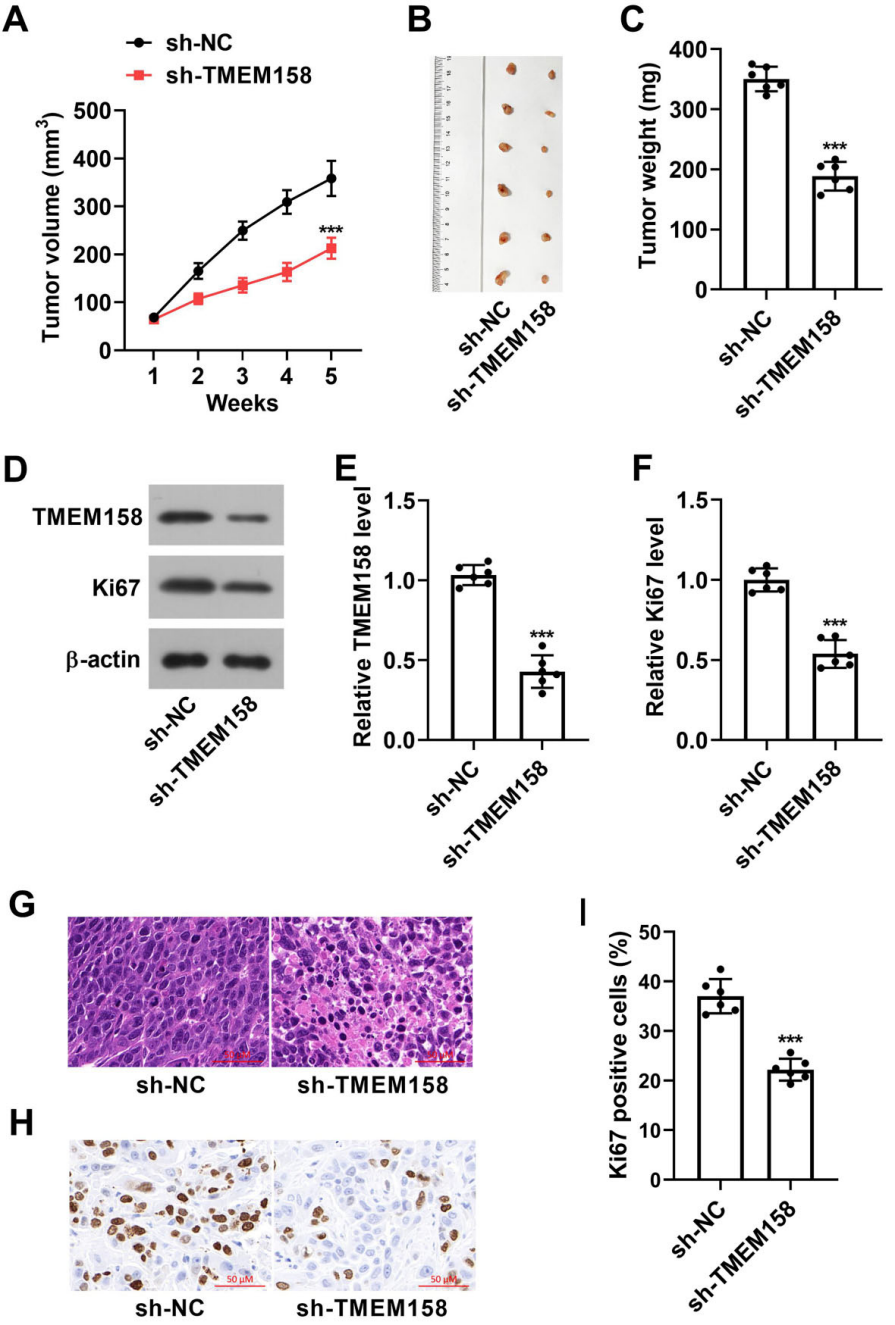
Lack of TMEM158 blocked tumor growth *in vivo*. **A**-**C**, Tumor volume and weight of the treated groups. **D**-**F**, TMEM158 and Ki67 levels were monitored by western blot. **G**, Representative photograph of HE staining; 400×magnification (scale bar 50 μM). **H**, Ki67 protein level was evaluated by IHC assay (400×magnification; scale bar 50 μM) and quantified (**I**). Data are reported as means±SD. ***P<0.001 (Student's *t*-test). NC: normal control.

To examine the effect of TMEM158 in GC, we introduced sh-TMEM158 or sh-NC-transfected AGS cells into mice. We found that downregulation of TMEM158 diminished tumor volume and weight compared with the sh-NC group (Figure 6A-C). The TMEM158 and Ki67 protein levels were decreased by sh-TMEM158 transfection (Figure 6D and F). In addition, the HE assay revealed that cancer cells were loosely arranged, and nuclei were pyknotic and fragmented in the sh-TMEM158 group (Figure 6G). Moreover, IHC assay also confirmed that Ki-67 content was reduced by silencing of TMEM158 (Figure 6H and I). These data demonstrated that the absence of TMEM158 inhibited GC tumorigenesis.

## Discussion

There are many factors leading to GC occurrence, such as *Helicobacter pylori* infection, smoking, alcohol consumption, high-salt diet, and genetics (2). At present, prevention and early detection are the main approaches to GC in the clinic. To prevent GC, experts advise people to change their unhealthy eating habits by eating more fresh fruits and vegetables, less salt, and less oil (1). People at high risk of gastric atrophy or ulceration need regular gastroscopic monitoring to facilitate early diagnosis (14). When GC is advanced, the patient may have continuous abdominal pain, inappetence, emaciation, hematemesis, and persistent vomiting. The lack of precise symptoms often leads to delay in diagnosis (1). We urgently need a diagnostic target to help doctors diagnose GC and determine patient prognosis. In this work, we primarily investigated the effect of TMEM158 in GC.

TMEM158 plays an imperative role in the prognosis and regulation of various cancers. Fu et al. (15) confirmed that the TMEM158 content was increased in pancreatic cancer (PC) samples and cells. TMEM158 upregulation was apparently associated with poorer prognosis of PC patients. In addition, Mohammed et al. (16) reported that lack of TMEM158 decreased the resistance to cisplatin in non-small cell lung cancer (NSCLC) cell and that TMEM158 could serve as a biomarker of cisplatin sensitivity in NSCLC. Furthermore, Li et al. (17) revealed that TMEM158 could act as a diagnostic marker for anaplastic thyroid carcinoma. Herein, we demonstrated that TMEM158 level was higher in GC tissues in the GSE and TCGA databases. Furthermore, the ROC curves displayed that the diagnostic value of TMEM158 content in GC was high. GC patients with a higher level of TMEM158 were associated with poor OS, which was similar to the findings of Fu et al. (15). In addition, TMEM158 content was increased in GC cells, which was in line with the results of GSE and TCGA database. We found that TMEM158 promoted GC cell proliferation and TMEM158 could promote activation of the PI3K/Akt signaling. Deficiency of TMEM158 suppressed GC tumor growth *in vivo*.

Previous articles proved that PI3K/AKT signaling was crucial for the progression of numerous cancers. Fu et al. (15) confirmed that TMEM158 could increase cell proliferation, cell cycle, and cell invasion by activation of PI3K/AKT signaling pathway in PC. In addition, Zhang et al. (18) revealed that a decrease of RNA m6A methylation facilitated PI3K/Akt signaling activation, which stimulated GC cell proliferation and invasion. Moreover, Wang et al. (19) also found that LINC01559 could contribute to GC progression via activation PI3K/AKT pathway. Furthermore, TREM2 could regulate GC cell proliferation and metastasis through the PI3K/AKT pathway (20). In this work, we discovered that PI3K activation or inhibition overturned the effect of TMEM158 silencing or overexpression on GC cell proliferation. This result meant that TMEM158 promoted GC cell proliferation through activating the PI3K/Akt signaling, which was similar to the findings of Fu et al. (15) and Zhang et al. (18). In this study, we demonstrated that TMEM158 could regulate the phosphorylation level of PI3K p85 alpha. Further research should be conducted to determine whether PI3K beta and PI3K delta are also regulated by TMEM158. Whether TMEM158 directly or indirectly modulates the PI3K function is unkonwn. If TMEM158 has inherent kinase activity, it is possible that TMEM158 directly phosphorylates PI3K and affects its function. If not, TMEM158 possibly indirectly modulates the PI3K function by regulating some kinases that increase the phosphorylation of PI3K.

The present study had several limitations. First, we did not investigate the underlying mechanism of the PI3K/AKT signal involved in TMEM158‐mediated malignant carcinogenesis. Second, we did not explore the other potential signaling pathways involved in the TMEM158‐induced GC progression. Other factors, including aging, animal species, and treatment protocol, also need to be considered to fully evaluate the oncogenic roles of TMEM158 in GC.

In summary, we established that TMEM158 content was increased in GC samples and cells. The high expression of TMEM158 was significant for GC diagnosis and linked with poor OS of GC patients. Furthermore, TMEM158 contributed to GC cell proliferation through modulating the PI3K/Akt signaling pathway and could serve as a target for GC treatment.
